# Growth performance of pigs fed low-protein diets supplemented with crystalline amino acids at different growth stages

**DOI:** 10.5713/ab.24.0339

**Published:** 2024-08-26

**Authors:** Inho Cho, Changsu Kong

**Affiliations:** 1 Department of Animal Science and Biotechnology, Kyungpook National University, Sangju 37224, Korea; 2 Research Institute for Innovative Animal Science, Kyungpook National University, Sangju 37224, Korea; 3 Department of Animal Science, Kyungpook National University, Sangju 37224, Korea

**Keywords:** Amino Acid, Low-protein Diet, Growth Performance, Growth Stage, Pig

## Abstract

**Objective:**

This study aimed to investigate the impact of reducing dietary crude protein (CP) coupled with supplementation of indispensable amino acids (AA) on growth performance of pigs at different growth stages.

**Methods:**

A total of 126 (63 barrows and 63 gilts), 90 (45 barrows and gilts), and 72 (36 barrows and 36 gilts) pigs with average weights of 9.8±1.62, 30.6±2.31, and 58.3±2.95 kg in the nursery, growing, and finishing stages, respectively, were assigned to three dietary treatments with six replicates in a randomized complete block design. The pigs had *ad libitum* access to water and fed three experimental diets, each supplemented with all indispensable AA and subjected to a 2% reduction in CP from the upper limits of 18%, 16%, and 16% established for the nursery, growing, and finishing stages, respectively.

**Results:**

In the nursery stage, from 0 to 2 weeks, reducing dietary CP concentrations decreased average daily feed intake (ADFI; linear, p = 0.04). From 2 to 4 weeks, dietary CP reduction decreased average daily gain (ADG; linear, p<0.01; quadratic, p = 0.02), ADFI (linear, p = 0.04), and gain-to-feed ratio (G:F; linear, p = 0.01). From 0 to 4 weeks, reduction in dietary CP concentrations decreased ADG (linear p<0.01), and G:F (linear, p = 0.01). In the growing stage, the dietary CP reduction did not affect growth performance. During the finishing stage, decrease in dietary CP concentrations decreased ADFI from 3 to 6 weeks (quadratic, p<0.01) and 0 to 6 weeks (quadratic, p = 0.01).

**Conclusion:**

Dietary CP reduction with indispensable AA supplementation potentially decreases the growth performance of nursery pigs but may not decrease the growth performance of growing and finishing pigs.

## INTRODUCTION

In pig production, dietary crude protein (CP) is a key factor indicating the requirement for amino acids (AA) rather than CP itself for protein synthesis [1]. To meet this need for AA, protein sources rich in CP, such as soybean meal (SBM), are commonly included in the diet [2,3]. The relatively large quantity of SBM has been included in practical diets because recommendation of the dietary CP level had been suggested high in the past [4]. Therefore, the inclusion of SBM in the traditional diet increased dietary CP concentration, leading to excess AA.

The traditional high-CP diets have certain shortcomings. The excess AA in the traditional high-CP diets increases nitrogen excretion due to undigested protein and unutilized AA. This excreted nitrogen is discharged into the soil and air as nitrogenous waste, such as ammonia and nitrous oxide, causing environmental pollution [5,6]. Additionally, when undigested protein reaches the hindgut, protein fermentation occurs and potentially impairs gut health in pigs [7,8]. Finally, the cost of SBM is anticipated to soar due to climate change [9] and increased demand resulting from elevated meat production [10]. Therefore, a low-protein (LP) diet with reduced dietary CP concentration has emerged as an effective strategy to address these problems.

The LP diets need to be supplemented with crystalline AA to satisfy requirements of pigs because dietary CP reduction could result in AA deficiency and imbalance. Supplementation of AA in LP diet leads to a more precise adjustment of AA supply to meet AA requirements. However, in previous research, the effect of LP diets supplemented with crystalline AA on growth performance was not consistent because the pattern of AA supplementation in each study was different [11]. As the dietary CP concentration decreases, more AA in LP diet may be restricted, potentially impairing the growth performance of pigs. Therefore, it seems necessary to investigate the impact of supplementing all indispensable AA in LP diet to satisfy the AA requirements on growth performance.

Additionally, the effect of LP diets on growth performance may vary depending on the growth stage. Despite variations in digestive ability, digestive organ development, and enzyme activity at different growth stages [12–14], information regarding the effect of LP diets on growth performance at each growth stage is limited. Therefore, this study aimed to investigate the effect of dietary CP reduction accompanied by supplementation with all indispensable AA on the growth performance of nursery, growing, and finishing pigs.

## MATERIALS AND METHODS

### Animal ethics

All the experimental procedures applied in this study were reviewed and approved by the Institutional Animal Care and Use Committee at Kyungpook National University, Republic of Korea (approval number: KNU 2023-0154).

### Animal trials and experimental design

Experiment 1 involved a total of 126 crossbred ([Yorkshire× Landrace]×Duroc) nursery pigs (63 barrows and 63 gilts), with an average weight of 9.8±1.62 kg. They were divided into 3 dietary treatments with 6 replicates in a randomized complete block design, considering initial body weight (BW) and sex as blocking factors. The pigs were grouped into pens, with 7 pigs per pen, and provided with ad libitum access to water and experimental diets for 4 weeks. They were housed in a controlled environment measuring 1.6 m×1.6 m, equipped with a feeder and water nipple. The experiment was comprised of 2 phases: 0 to 2 weeks and 2 to 4 weeks. Room temperature was maintained at 30°C in the first week, decreased by 1°C every week.

Experiment 2 was conducted with a total of 90 crossbred ([Yorkshire×Landrace]×Duroc) growing pigs, comprising 45 barrows and 45 gilts, averaging 30.6±2.31 kg in weight. Pigs were allocated to 3 dietary treatments with 6 replicates in a randomized complete block design, considering initial BW and sex as blocking factors. Pigs were grouped into pens, with 5 pigs per pen and provided with ad libitum access to water and experimental diets, for 5 weeks. They were reared in an environmentally controlled facility (2.60 m×2.84 m) equipped with a feeder and water nipple. The experiment has 2 phases: 0 to 3 weeks and 3 to 5 weeks. Room temperature in the experimental house was maintained at 26°C.

Experiment 3 was conducted with a total of 72 crossbred ([Yorkshire×Landrace]×Duroc) finishing pigs, consisting of 36 barrows and 36 gilts, with an average weight of 58.3±2.95 kg. Pigs were assigned to 3 dietary treatments with 6 replicates in a randomized complete block design, considering initial BW and sex as blocking factors. The pigs were sorted into groups of 4 pigs per pen and provided with ad libitum access to water and experimental diets, throughout the 6 weeks period. Pigs were reared in an environmentally controlled facility (2.60 m×2.84 m) equipped with a feeder and water nipple. The experiment was divided into 2 phases: 0 to 3 weeks and 3 to 6 weeks. Room temperature in the experimental house was controlled stably at 22°C for the 6 weeks.

All experiments were conducted at the experimental farm of Seoul National University.Individual BW and feed leftover in all experiments were recorded at each phase, and average daily gain (ADG), average daily feed intake (ADFI), and gain-to-feed (G:F) were calculated.

### Dietary treatments

The dietary treatments comprised 3 diets, each of which replaced the SBM content with corn and crystalline AA, resulting in a 2% reduction in dietary CP concentrations from the upper limit specified for each growth stage (Tables 1, 2, and 3). The upper limits of dietary CP levels for the nursery, growing and finishing stages were set at 18%, 16%, and 16%, respectively. All experimental diets were formulated to meet or exceed the nutrient requirements of nursery, growing, and finishing pigs according to NRC [15]. The chemical and analyzed chemical compositions of the experimental diets are presented in Tables 4 and 5. All experimental diets were manufactured at Dodram B&F Mill (Eumseong, Korea).

### Chemical analysis

The experimental diets were ground using a mill grinder (CT 293 Cyclotec; Foss Ltd., Hillerød, Denmark) for analyses. They were analyzed for AA content (method 994.12) as described in AOAC [16]. The CP, calcium, and phosphorus contents of the experimental diets were analyzed (method 968.08; method 965.17) as described in AOAC [16].

### Data analysis

Data were analyzed using the MIXED procedure of SAS software (SAS 9.4, SAS Inst. Inc., Cary, NC, USA). Dietary treatment was regarded as a fixed variable, while the replicate was considered a random variable. The experimental unit was a pen. Linear and quadratic effects of dietary treatment were identified using orthogonal polynomial contrasts, and statistical significance was set at p<0.05.

## RESULTS

There is a gap between calculated nutrients (Table 4) and the analyzed nutrients (Table 5) of experimental diets, during nursery stage. The growth performance of nursery pigs is presented in Table 6. Body weight decreased linearly (p = 0.01) with dietary CP reduction at week 4. Linear (p<0.01) and quadratic (p = 0.02) decreases in the ADG of pigs fed LP diets were observed from 2 to 4 weeks. From 0 to 4 weeks, ADG both linearly (p<0.01) and quadratically (p = 0.05) decreased as dietary CP reduced from 18% to 14%. A linear response (p = 0.04) in ADFI occurred as dietary CP decreased from 0 to 2 weeks and 2 to 4 weeks. Dietary CP reduction linearly decreased G:F from 2 to 4 weeks (p = 0.01) and 0 to 4 weeks (p<0.01). As shown in Table 7, reducing the concentration of dietary CP had no effect on the growth performance of growing pigs, regardless of phase. However, as shown in Table 8, dietary CP reduction exerted a quadratic effect on ADFI from 3 to 6 weeks (p<0.01) and 0 to 6 weeks (p = 0.01) but had no effect on BW, ADG, and G:F.

## DISCUSSION

The calculated values of experimental diets differed from the analyzed, with the deviation of lysine being particularly large during the nursery stage. The gap may be attributed to measurement error and mixing error. Although the feed ingredients in this experiment 1 were same, the variation in the analyzed values of the experimental diets were different, which may suggest that the mixability was not constant.

Low-protein diets supplemented with indispensable AA have been shown to decrease occurrences of diarrhea in nursery pigs [17] and N excretion [18], and improve gut health [7]. These LP diets supplemented with indispensable AA have been anticipated to maintain the growth performance of pigs. Mavromichalis et al [19] demonstrated that an LP diet supplemented with indispensable AA facilitated the reduction of dietary CP from 19.2% to 13.5% without compromising nursery pig growth performance, possibly because their dietary AA requirements had been met through AA supplementation. In experiment 1, the growth performance of nursery pigs decreased with decreasing dietary CP levels, especially from 16% to 14%, despite supplementation with indispensable AA. The discrepancy between the current and previous studies may be ascribed to differences in dietary AA concentration and ingredient compositions. Unlike the current study, Mavromichalis et al [19] fortified LP diets with indispensable AA to reach 110% of the AA requirement. Although the CP concentrations in each LP diet were similar, differences in dietary indispensable AA concentrations may explain these contradictory results. Additionally, dietary SBM inclusion in a CP 13.5% diet in Mavromichalis et al [19] was 13.5%, whereas the dietary SBM inclusion was 2% in the CP 14% diet in the present study as blood plasma and fish meal were incorporated in the diet. According to NRC [15], the ratio of standardized ileal digestible (SID) total dispensable AA to SID CP in SBM was greater than in blood plasma and fish meal. Therefore, the CP 13.5% diet containing greater SBM in the study of Mavromichalis et al [19] might contain higher dispensable AA concentrations potentially available for pigs, compared to the diet in the present study. Another possibility is lower analyzed lysine concentrations than requirement in CP 14% diet could contribute to decreases in ADG and G:F, which may explain the discrepancy between the current and previous studies. Therefore, precise diet formulation is crucial for LP diet research.

In contrast, Nyachoti et al [20] reported a decrease in the growth performance of nursery pigs fed LP diets supplemented with indispensable AA, which is consistent with the findings of the current study. The impaired growth performance may be attributed to the restriction of protein synthesis resulting from reduced concentrations of dispensable AA and diminishing synthesis of dispensable AA due to minimizing the excess supply of indispensable AA. In experiment 1, based on the CP 18% diet, the mean relative values of all dispensable AA concentrations in the CP 16% and CP 14% diets were 98.5% and 73.8%, respectively. This drastic decrease in dispensable AA in the CP 14% diet potentially supports the quadratic decrease in ADG. An adequate supply of AA, including dispensable AA, is necessary for protein synthesis [21]. Furthermore, dispensable AA are also required for organ improvement and enhancement of protein synthesis [22]. Further research is warranted to determine the extent to which dispensable AA concentrations can be reduced by supplementation with indispensable AA without affecting growth performance.

In experiment 2, the reduction in dietary CP from 16% to 12% accompanied by supplementation with indispensable AA did not affect the growth performance of growing pigs. In contrast to our study, some studies reported that LP diets supplemented with indispensable AA resulted in a decrease in growth performance of growing pigs as dietary CP reduced [23–25]. A difference between aforementioned and current studies is the AA supplementation pattern. In previous studies, it was common practice to supplement lysine, methionine, threonine, and tryptophan, but in this case, there is a possibility that the remaining indispensable AA might be insufficient. However, in the current study, all indispensable AA were supplemented to meet all AA requirements of pigs. On the other hand, other studies that reduced the dietary CP concentrations in a high-CP control diet by more than 4% units and supplemented it with all indispensable AA to satisfy all AA requirements demonstrated that an LP diet supplemented with AA did not affect the growth performance of growing pigs [26–28], supporting that the AA supplementation pattern may affect the growth performance. This also suggests that all AA in the diets of the current study, including dispensable AA, may not be limited for growth performance of growing pigs. Additionally, in experiment 2, based on the CP 16% diet, the mean relative values of all dispensable AA concentrations in the CP 14% and CP 12% diets were 81.1% and 63.1%, respectively which were not consistent with experiment 1. According to the NRC [15], the dietary CP requirements, based on total nitrogen content, for pigs weighing 10 to 30 kg and 30 to 60 kg were calculated to be 18.3% and 15.1%, respectively. This indicates that the CP 16% diet may include sufficient dispensable AA for growing pigs, and dispensable AA may not be deficient when dietary CP concentrations are above 12% during the growing stage.

In experiment 3, the reduction in dietary CP from 16% to 12% accompanied by supplementation with indispensable AA did not affect the growth performance of finishing pigs, except for ADFI. In contrast, Souza et al [29] reported that the dietary CP reduction from 19% to 13.2%, accompanied by supplementation with lysine, methionine, threonine, and tryptophan, decreased the growth performance of finishing pigs. Nevertheless, Song et al [30] reported that reducing dietary CP from 15% to 12.6% and concurrently supplementing with indispensable AA to satisfy all AA requirements did not affect the growth performance of finishing pigs, suggesting that balanced supplementation with indispensable AA is necessary for maintaining growth performance. Furthermore, in experiment 3, based on the CP 16% diet, the mean relative values of all dispensable AA concentrations in the CP 14% and CP 12% diets were 85.9% and 78.7%, respectively. These results are consistent with experiment 2. According to the NRC [15], the dietary CP requirement, based on total nitrogen content, for pigs weighing 60 to 100 kg was calculated to be 12.7%. This indicates that the CP 16% diet may contain sufficient concentrations of dietary dispensable AA for finishing pigs, and dietary dispensable AA concentrations may not be deficient when the dietary CP concentration is above 12%, even during the finishing stage. Regarding ADFI, the major factors that affect the feed intake of pigs are dietary energy concentration and AA balance [31]. In this study, however, differences in dietary energy concentration among treatments were negligible, and dietary AA were well balanced, as excess AA were minimized through supplementation with all indispensable AA to meet all AA requirements for pigs. Further research is needed to elucidate the effect of LP diets on the feed intake of pigs.

The impact of LP diets on the growth performance of pigs varied across different growth stages. This variability may be attributed to the developmental status depending on the growth stage. During the nursery stage, visceral organs such as the liver, pancreas, and kidneys, which play crucial roles in utilizing and metabolizing nutrients, appear to develop relatively rapidly [13–14]. Moreover, enzyme activity in visceral organs increases during the nursery stage as these organs and offal develop. Sun et al [32] reported that intestinal morphology, as well as the development of the liver and kidneys, were impaired in nursery pigs fed a CP 14% diet supplemented with indispensable AA compared to those fed a CP 20% diet. Additionally, the mRNA expression level of the AA transporter in nursery pigs fed a CP 14% diet was correspondingly lower than that of those fed a CP 20% diet. However, Li et al [33] found that an LP diet supplemented with indispensable AA did not reduce the mRNA expression of AA transporters in growing pigs. The LP diets supplemented with indispensable AA potentially exert varying effects on organ development and enzyme function depending on the growth stage. As a result, their effects on the growth performance of pigs may also vary with the growth stage. Therefore, considering this variability is essential when conducting further research on the effect of LP diets on the growth performance of pigs.

## CONCLUSION

Dietary reduction in CP accompanied by supplementation with all indispensable AA to satisfy all AA requirements for pigs exhibits varying effects on growth performance depending on the growth stage. Reduction of the dietary CP, particularly down to 12%, appears feasible during the growing and finishing stages, without adverse effects on growth performance. However, in this study, supplementation with indispensable AA in a LP diet potentially reduces the growth performance due to indispensable or dispensable AA deficiency during the nursery stage. Further research is needed to determine the accurate dietary CP concentrations at which supplementation with all indispensable AA does not negatively affect the growth performance of pigs and whether further reduction in the dietary CP concentrations can be achieved with supplementation with dispensable AA.

## Figures and Tables

**Table 1:** Ingredient compositions of experimental diets for nursery pigs (as-fed basis)

Item	Nursery		

	CP 18%	CP 16%	CP 14%
Ingredients (%)			
Corn	66.67	72.10	77.52
Dehulled SBM	15.20	8.60	2.00
Fish meal	4.00	4.00	4.00
Blood plasma	2.00	2.00	2.00
Milk whey powder	8.00	8.00	8.00
L-Lysine-HCl	0.76	1.10	1.43
L-Threonine	0.15	0.25	0.34
DL-Methionine	0.18	0.25	0.31
L-Tryptophan	0.02	0.06	0.10
L-Isoleucine	0.02	0.14	0.26
L-Valine	0.06	0.18	0.29
L-Phenylalanine	0.01	0.14	0.27
L-Histidine	0.02	0.09	0.15
L-Leucine	-	0.11	0.22
L-Arginine	-	0.01	0.01
Beef tallow	1.00	1.00	1.00
Sodium chloride	0.20	0.20	0.20
Limestone	1.10	1.12	1.13
Dicalcium phosphate	0.40	0.48	0.56
Phytase	0.01	0.01	0.01
Vitamin premix^1)^	0.10	0.10	0.10
Mineral premix^2)^	0.10	0.10	0.10
Total	100.00	100.00	100.00

CP, crude protein; SBM, soybean meal.

1) Provided per kilogram diet: vitamin A, 11,000,000 IU; vitamin D, 920,000 IU; vitamin E, 65,000 mg; vitamin K_3_, 7,500; vitamin B_1_, 2,800 mg; vitamin B_2_, 8,500 mg; vitamin B_6_, 4,000 mg; vitamin B_12_, 45 mg; biotin, 190 mg; calcium pantothenate, 37,000 mg; folic acid, 550 mg; and niacin, 55,000 mg.

2) Provided per kilogram diet: Mn, 30,000 mg; Cu, 15,000 mg; Zn, 30,000 mg; Fe, 75,000 mg; I, 250 mg; Co, 250 mg; and Se, 100 mg.

**Table 2:** Ingredient compositions of experimental diets for growing pigs (as-fed basis)

Item	Grower		

	CP 16%	CP 14%	CP 12%
Ingredients (%)			
Corn	70.91	76.67	82.42
SBM	16.20	9.45	2.70
Corn DDGS	4.50	4.50	4.50
Canola meal	4.00	4.00	4.00
L-Lysine-HCl	0.77	1.08	1.39
L-Threonine	0.13	0.22	0.31
DL-Methionine	0.09	0.16	0.22
L-Tryptophan	0.03	0.07	0.10
L-Isoleucine	-	0.11	0.21
L-Valine	0.05	0.16	0.26
L-Phenylalanine	-	0.10	0.19
L-Histidine	-	0.05	0.10
L-Leucine	-	0.04	0.07
Beef tallow	1.00	1.00	1.00
Sodium chloride	0.30	0.30	0.30
Limestone	0.97	0.97	0.97
Dicalcium phosphate	0.84	0.95	1.05
Phytase	0.01	0.01	0.01
Vitamin premix^1)^	0.10	0.10	0.10
Mineral premix^2)^	0.10	0.10	0.10
Total	100.00	100.00	100.00

CP, crude protein; SBM, soybean meal; DDGS, distiller’s dried grains with soluble.

1) Provided per kilogram diet: vitamin A, 8,000,000 IU; vitamin D, 800,000 IU; vitamin E, 40,000 mg; vitamin K_3_, 4,000; vitamin B_1_, 2,000 mg; vitamin B_2_, 9,200 mg; vitamin B_6_, 3,000 mg; vitamin B_12_, 30 mg; biotin, 200 mg; calcium pantothenate, 20,000 mg; folic, 600 mg; and niacin, 50,000 mg.

2) Provided per kilogram diet: Mn, 40,000 mg; Cu, 20,000 mg; Zn, 51,000 mg; Fe, 80,000 mg; I, 450 mg; Co, 500 mg; and Se, 200 mg.

**Table 3:** Ingredient compositions of experimental diets for finishing pigs (as-fed basis)

Item	Finisher		

	CP 16%	CP 14%	CP 12%
Ingredients (%)			
Corn	69.80	75.33	80.95
SBM with hulls	14.60	8.50	2.00
Corn DDGS	6.00	6.00	6.00
Canola meal	6.00	6.00	6.00
L-Lysine-HCl	0.52	0.80	1.10
L-Threonine	0.05	0.13	0.21
DL-Methionine	-	0.05	0.11
L-Tryptophan	0.01	0.04	0.07
L-Isoleucine	-	0.02	0.13
L-Valine	-	0.03	0.13
L-Phenylalanine	-	-	0.08
L-Histidine	-	-	0.05
Beef tallow	1.00	1.00	1.00
Sodium chloride	0.30	0.30	0.30
Limestone	0.97	0.97	0.97
Dicalcium phosphate	0.54	0.62	0.69
Phytase	0.01	0.01	0.01
Vitamin premix^1)^	0.10	0.10	0.10
Mineral premix^2)^	0.10	0.10	0.10
Total	100.00	100.00	100.00

CP, crude protein; SBM, soybean meal; DDGS, distiller’s dried grains with soluble.

1) Provided per kilogram diet: vitamin A, 8,000,000 IU; vitamin D, 800,000 IU; vitamin E, 40,000 mg; vitamin K_3_, 4,000; vitamin B_1_, 2,000 mg; vitamin B_2_, 9,200 mg; vitamin B_6_, 3,000 mg; vitamin B_12_, 30 mg; biotin, 200 mg; calcium pantothenate, 20,000 mg; folic, 600 mg; and niacin, 50,000 mg.

2) Provided per kilogram diet: Mn, 40,000 mg; Cu, 20,000 mg; Zn, 51,000 mg; Fe, 80,000 mg; I, 450 mg; Co, 500 mg; and Se, 200 mg.

**Table 4:** Calculated nutrient and chemical compositions of experimental diets for pigs (as-fed basis)

Item (%)	Nursery			Grower			Finisher		
		
	CP 18%	CP 16%	CP 14%	CP 16%	CP 14%	CP 12%	CP 16%	CP 14%	CP 12%
ME (kcal/kg)	3,391	3,396	3,401	3,355	3,357	3,359	3,351	3,356	3,360
CP	18.00	16.00	13.99	16.00	13.99	11.97	16.12	14.12	12.20
Ca	0.70	0.70	0.70	0.66	0.66	0.66	0.59	0.59	0.59
P	0.48	0.46	0.45	0.49	0.48	0.47	0.46	0.45	0.43
SID AA									
Arginine	0.94	0.75	0.56	0.82	0.64	0.47	0.83	0.67	0.49
Histidine	0.42	0.42	0.42	0.36	0.35	0.34	0.37	0.31	0.29
Isoleucine	0.63	0.63	0.63	0.52	0.51	0.51	0.52	0.45	0.45
Leucine	1.35	1.29	1.23	1.24	1.11	0.99	1.27	1.13	0.99
Lysine	1.23	1.23	1.23	0.98	0.98	0.98	0.85	0.85	0.85
Methionine	0.44	0.47	0.50	0.32	0.35	0.38	0.24	0.26	0.29
Met+Cys	0.68	0.68	0.68	0.55	0.55	0.55	0.48	0.48	0.48
Phenylalanine	0.72	0.72	0.72	0.64	0.61	0.59	0.64	0.54	0.51
Threonine	0.73	0.73	0.73	0.59	0.59	0.59	0.52	0.52	0.52
Tryptophan	0.20	0.20	0.20	0.17	0.17	0.17	0.15	0.15	0.15
Valine	0.78	0.78	0.78	0.64	0.64	0.64	0.61	0.55	0.55

CP, crude protein; ME, metabolizable energy; Ca, calcium; P, phosphorous; SID, standardized ileal digestible; AA, amino acid; Met+Cys, methionine+cysteine.

**Table 5:** Analyzed nutrient and chemical compositions of experimental diets for pigs (as-fed basis)

Item (%)	Nursery			Grower			Finisher		
		
	CP 18%	CP 16%	CP 14%	CP 16%	CP 14%	CP 12%	CP 16%	CP 14%	CP 12%
CP	17.63	17.10	14.55	17.34	15.12	12.50	16.41	13.78	12.50
Ca	0.69	0.71	0.77	0.77	0.78	0.65	0.56	0.53	0.85
P	0.42	0.44	0.44	0.53	0.49	0.44	0.44	0.42	0.49
Indispensable AA									
Arginine	1.00	0.96	0.66	0.99	0.75	0.53	0.89	0.72	0.61
Histidine	0.47	0.48	0.44	0.46	0.41	0.37	0.41	0.35	0.37
Isoleucine	0.70	0.70	0.65	0.65	0.59	0.54	0.57	0.48	0.52
Leucine	1.57	1.58	1.39	1.60	1.37	1.16	1.48	1.31	1.22
Lysine	1.27	1.27	1.10	1.18	1.09	1.01	0.93	0.86	1.00
Methionine	0.43	0.45	0.47	0.31	0.40	0.35	0.26	0.26	0.33
Phenylalanine	0.83	0.84	0.77	0.82	0.73	0.66	0.72	0.61	0.61
Threonine	0.89	0.86	0.80	0.80	0.77	0.68	0.65	0.60	0.67
Valine	0.91	0.91	0.86	0.85	0.80	0.75	0.73	0.67	0.72
Dispensable AA									
Alanine	0.94	0.94	0.76	0.94	0.79	0.65	0.87	0.78	0.73
Aspartic acid	1.57	1.55	1.03	1.51	1.11	0.77	1.28	1.03	0.84
Cysteine	0.32	0.31	0.26	0.32	0.29	0.24	0.32	0.29	0.28
Glutamic acid	2.77	2.75	2.00	2.99	2.41	1.86	2.72	2.33	2.12
Glycine	0.74	0.74	0.56	0.71	0.55	0.42	0.62	0.54	0.49
Proline	1.08	1.08	0.88	1.15	1.02	0.88	1.13	1.03	1.02
Serine	0.83	0.81	0.60	0.82	0.66	0.50	0.74	0.62	0.57
Tyrosine	0.63	0.57	0.46	0.55	0.46	0.35	0.53	0.43	0.41
Mean relative values^1)^	100	98.5	73.8	100	81.1	63.1	100	85.9	78.7

CP, crude protein; Ca, calcium; P, phosphorous; AA, amino acid.

1) Mean relative values of dispensable AA based on CP 18%, CP 16%, and CP 16% diet in the nursery, grower, and finisher stages, respectively.

**Table 6:** Growth performance of nursery pigs fed experimental diets

Item	Dietary treatments			SEM	p-value	
	
	CP 18%	CP 16%	CP 14%		Linear^1)^	Quadratic^2)^
BW (kg)						
At wk 0	9.8	9.8	9.8	0.64	1.00	0.66
At wk 2	14.5	14.3	14.3	1.01	0.60	0.86
At wk 4	22.5	22.6	20.6	1.51	0.01	0.07
ADG (g)						
0 to 2 wk	328.1	321.4	323.5	27.97	0.82	0.80
2 to 4 wk	573.7	592.9	451.0	39.90	<0.01	0.02
0 to 4 wk	450.9	457.2	387.3	32.01	<0.01	0.05
ADFI (g)						
0 to 2 wk	548.1	564.8	599.8	40.50	0.04	0.64
2 to 4 wk	995.4	1,016.5	893.9	62.11	0.04	0.08
0 to 4 wk	771.8	790.7	746.8	50.46	0.42	0.25
G:F (g/kg)						
0 to 2 wk	591.9	569.0	540.5	20.46	0.10	0.91
2 to 4 wk	579.4	584.0	498.1	19.94	0.01	0.08
0 to 4 wk	584.5	578.8	514.9	15.71	<0.01	0.14

CP, crude protein; SEM, standard error of the mean; BW, body weight; ADG, average daily gain; ADFI, average daily feed intake; G:F, gain to feed ratio.

1) Linear effect of dietary crude protein concentrations on growth performance.

2) Quadratic effect of crude protein concentrations on growth performance.

**Table 7:** Growth performance of growing pigs fed experimental diets

Item	Dietary treatments			SEM	p-value	
	
	CP 16%	CP 14%	CP 12%		Linear^1)^	Quadratic^2)^
BW (kg)						
At wk 0	30.6	30.6	30.6	0.96	0.64	0.30
At wk 3	45.2	44.1	44.1	2.40	0.51	0.82
At wk 5	56.9	55.5	54.1	3.21	0.23	0.82
ADG (g)						
0 to 3 wk	697.8	643.0	643.3	77.55	0.48	0.90
3 to 5 wk	832.0	817.2	714.0	65.68	0.10	0.46
0 to 5 wk	751.5	712.7	671.6	69.64	0.22	0.77
ADFI (g)						
0 to 3 wk	1,655.6	1,647.7	1,602.4	144.61	0.79	0.46
3 to 5 wk	2,282.6	2,183.2	2,000.7	201.22	0.27	0.70
0 to 5 wk	1,906.4	1,861.8	1,761.7	163.65	0.57	0.58
G:F (g/kg)						
0 to 3 wk	420.5	386.3	400.6	30.58	0.22	0.31
3 to 5 wk	364.1	376.9	360.2	22.19	0.26	0.45
0 to 5 wk	393.6	381.2	382.0	25.17	0.16	0.62

CP, crude protein; SEM, standard error of the mean; BW, body weight; ADG, average daily gain; ADFI, average daily feed intake; G:F, gain to feed ratio.

1) Linear effect of crude protein concentrations on growth performance.

2) Quadratic effect of crude protein concentrations on growth performance.

**Table 8:** Growth performance of finishing pig fed experimental diets.

Item	Dietary treatments			SEM	p-value	
	
	CP 16%	CP 14%	CP 12%		Linear^1)^	Quadratic^2)^
BW (kg)						
At wk 0	58.3	58.4	58.4	1.20	0.83	0.90
At wk 3	79.5	79.0	80.0	1.54	0.83	0.70
At wk 6	101.2	102.5	103.5	2.08	0.36	0.96
ADG (g)						
0 to 3 wk	1,007.6	1,023.8	1,030.0	56.78	0.78	0.94
3 to 6 wk	1,032.9	1,072.5	1,109.5	56.59	0.33	0.98
0 to 6 wk	1,020.2	1,048.2	1,074.6	40.30	0.36	0.99
ADFI (g)						
0 to 3 wk	2,559.5	2,621.6	2,455.3	87.60	0.36	0.25
3 to 6 wk	2,728.2	3,149.5	2,866.5	104.51	0.28	<0.01
0 to 6 wk	2,643.8	2,885.6	2,650.0	83.08	0.95	0.01
G:F (g/kg)						
0 to 3 wk	395.2	390.7	420.0	20.07	0.40	0.50
3 to 6 wk	380.2	341.8	388.0	20.11	0.79	0.12
0 to 6 wk	386.8	364.3	405.3	13.87	0.36	0.08

CP, crude protein; SEM, standard error of the mean; BW, body weight; ADG, average daily gain; ADFI, average daily feed intake; G:F, gain to feed ratio.

1) Linear effect of crude protein concentrations on growth performance.

2) Quadratic effect of crude protein concentrations on growth performance.

## References

[1] Gloaguen M, Le Floc’h N, Corrent E, Primot Y, van Milgen J (2014). The use of free amino acids allows formulating very low crude protein diets for piglets. J Anim Sci.

[2] Florou-Paneri P, Christaki E, Giannenas I (2014). Alternative protein sources to soybean meal in pig diets. J Food Agric Environ.

[3] Giuliana P, Francesca T, Riccardo F (2020). Protein hunger of the feed sector: the alternatives offered by the plant world. Ital J Anim Sci.

[4] NRC (1998). Nutrient requirements of swine.

[5] Dourmad JY, Jondreville C (2007). Impact of nutrition on nitrogen, phosphorus, Cu and Zn in pig manure, and on emissions of ammonia and odours. Livest Sci.

[6] Cappelaere L, Grandmaison JLC, Martin N, Lambert W (2021). Amino acid supplementation to reduce environmental impacts of broiler and pig production: a review. Front Vet Sci.

[7] Jha R, Berrocoso JFD (2016). Dietary fiber and protein fermentation in the intestine of swine and their interactive effects on gut health and on the environment: a review. Anim Feed Sci Technol.

[8] Pluske JR, Pethick DW, Hopwood DE, Hampson DJ (2002). Nutritional influences on some major enteric bacterial diseases of pig. Nutr Res Rev.

[9] Wheeler T, Reynolds C (2013). Predicting the risks from climate change to forage and crop production for animal feed. Anim Front.

[10] (c2012). World agriculture towards2030/2050: the 2012 revision [Internet].

[11] Wang Y, Zhou J, Wang G, Cai S, Zeng X, Qiao S (2018). Advances in low-protein diets for swine. J Anim Sci Biotechnol.

[12] Corring T, Aumaitre A, Durand G (1978). Development of digestive enzymes in the piglet from birth to 8 weeks: I. pancreas and pancreatic enzymes. Nutr Metab.

[13] Elefson SK, Lu N, Chevalier T (2021). Assessment of visceral organ growth in pigs from birth through 150 kg. J Anim Sci.

[14] McConnell JC, Barth KM, Griffin SA (1971). Nutrient digestibility and nitrogen metabolism studies at different stages of growth with fat and lean type swine fed two levels of protein. J Anim Sci.

[15] NRC (2012). Nutrient requirements of swine.

[16] AOAC (2016). Official methods of analysis of AOAC International.

[17] Rezaei R, Knabe DA, Tekwe CD (2013). Dietary supplementation with monosodium glutamate is safe and improves growth performance in postweaning pigs. Amino Acids.

[18] Tuitoek K, Young LG, de Lange CFM, Kerr BJ (1997). The effect of reducing excess dietary amino acids on growing-finishing pig performance: an elevation of the ideal protein concept. J Anim Sci.

[19] Mavromichalis I, Webel DM, Emmert JL, Moser RL, Baker DH (1998). Limiting order of amino acids in a low-protein corn-soybean meal-whey-based diet for nursery pigs. J Anim Sci.

[20] Nyachoti CM, Omogbenigun FO, Rademacher M, Blank G (2006). Performance responses and indicators of gastrointestinal health in early-weaned pigs fed low-protein amino acid-supplemented diets. J Anim Sci.

[21] Wu G (2014). Dietary requirements of synthesizable amino acids by animals: a paradigm shift in protein nutrition. J Anim Sci Biotechnol.

[22] Deng D, Yao K, Chu W (2009). Impaired translation initiation activation and reduced protein synthesis in weaned piglets fed a low-protein diet. J Nutr Biochem.

[23] Fang LH, Jin YH, Do SH (2019). Effects of dietary energy and crude protein levels on growth performance, blood profiles, and nutrient digestibility in weaning pigs. Asian-Australas J Anim Sci.

[24] He L, Wu L, Xu Z (2015). Low-protein diets affect ileal amino acid digestibility and gene expression of digestive enzymes in growing and finishing pigs. Amino Acids.

[25] Le Bellego L, van Milgen J, Noblet J (2002). Effect of high temperature and low-protein diets on the performance of growing-finishing pigs. J Anim Sci.

[26] Morales A, Buenabad L, Castillo G (2020). Serum concentration of free amino acids in pigs of similar performance fed diets containing protein-bound or protein-bound combined with free amino acids. Anim Feed Sci Technol.

[27] Yi XW, Zhang SR, Yang Q, Yin HH, Qiao SY (2010). Influence of dietary net energy content on performance of growing pigs fed low crude protein diets supplemented with crystalline amino acids. J Swine Health Prod.

[28] Zhao Y, Tian G, Chen D (2019). Effect of different dietary protein levels and amino acids supplementation patterns on growth performance, carcass characteristics and nitrogen excretion in growing-finishing pigs. J Anim Sci Biotechnol.

[29] Souza FNC, Genova JL, Gregory CR (2023). Low- and high-protein diets supplemented up to the fourth limiting amino acid for genetic lines of grower-finisher pigs. Livest Sci.

[30] Song W, Wu Z, Li W, Li Y (2023). Multiple amino acid supplementations to reduce dietary protein for pigs during early and late finisher periods under commercial conditions. J Sci Food Agric.

[31] Li Q, Patience JF (2017). Factors involved in the regulation of feed and energy intake of pigs. Anim Feed Sci Technol.

[32] Sun Y, Teng T, Bai G (2020). Protein-restricted diet balanced for lysine, methionine, threonine, and tryptophan for nursery pigs elicits subsequent compensatory growth and has long term effects on protein metabolism and organ development and tryptophan for nursery pigs elicits subsequent compensatory growth and has long term effects on protein metabolism and organ development. Anim Feed Sci Technol.

[33] Li Y, Wei H, Li F (2016). Supplementation of branched-chain amino acids in protein-restricted diets modulates the expression levels of amino acid transporters and energy metabolism associated regulators in the adipose tissue of growing pigs. Anim Nutr.

